# Cross-cultural validation of stool Based Colorectal cancer screening methods in the North West of Iran

**DOI:** 10.1016/j.amsu.2022.103494

**Published:** 2022-03-12

**Authors:** Roya Dolatkhah, Mohammad Hossein Somi, Saeed Dastgiri, Mohammad Asghari Jafarabadi, Hossein Mashhadi Abdolahi, Dariush Shanehbandi, Milad Asadi, Marzieh Nezamdoust, Neda Dolatkhah, Faris Farassati

**Affiliations:** a Hematology and Oncology Research Center, Tabriz University of Medical Sciences, Tabriz, Iran; b Liver and Gastrointestinal Diseases Research Center, Tabriz University of Medical Sciences, Tabriz, Iran; c Tabriz Health Services Management Research Center, Tabriz University of Medical Sciences, Tabriz, Iran; d Dept. Of Statistics and Epidemiology, School of Medicine, Zanjan University of Medical Sciences, Zanjan, Iran; e Immunology Research Center, Tabriz University of Medical Sciences, Tabriz, Iran; f Department of Basic Oncology, Health Institute of Ege University, Izmir, Turkey; g Tabriz University of Medical Sciences, Tabriz, Iran; h Physical Medicine and Rehabilitation Research Center, Aging Research Institute, Tabriz University of Medical Sciences, Tabriz, Iran; i Midwest Biomedical Research Foundation, Kansas City, MO, USA

**Keywords:** Colorectal cancer, Screening, Diagnostic accuracy, Colonoscopy, Stool based test

## Abstract

**Background:**

Globally, colorectal cancer (CRC) is the third most common cancer and the second leading cause of death from cancer. Incidence and mortality from CRCboth can be reduced and prevented using screening and early detection programs. The current study aimed to assess the feasibility of the colorectal cancer screening program in Northwest of Iran.

**Methods:**

The study designed as a cross-cultural analytic study, to evaluate the diagnostic accuracy of stool-based tests compared with colonoscopy, during 2016–2020. All individuals first were assessed with our CRC risk assessment tool, then eligible volunteers entered the study. Colonoscopy was performed on all participants, also stool-based tests including traditional guaiac, high-sensitivity guaiac-based, fecal immunochemical test (FIT), and multitarget stool DNA (Mt-sDNA) panel tests were performed.

**Results:**

Mt-sDNA test panel had a sensitivity of 77.8% (95% CI: 40–97.2)for detecting colorectal cancer with a specificity of 91.2% (95% CI:85.4–95.2). The FIT test alone had a lower sensitivity (66.7%; 95% CI:29.9–92.5) and almost the same specificity of 93.9% (95% CI: 88.7–97.2) for cancer detection. Mt-sDNA test had better diagnostic accuracy than the FIT (AUC = 0.85 vs 0.80), and is a more useful screening test. Positive and negative predictive values for cancer detection for both Mt-sDNA and FIT tests were almost the same results, however Mt-sDNA test had better NPV results than the FIT test alone.

**Conclusion:**

Our results showed that both Mt-sDNA panel and the FIT test had acceptable cut-off points for cancer detection, however, Mt-sDNA test had better diagnostic accuracy.

## Highlights

• A priority of Iranian health policy must be to develop an executive plan to identify the appropriate CRC screening method.
• This study provides a first step toward the design of a population-based CRC screening program in East Azerbaijan, Iran.
• The current study has been performed to evaluate the diagnostic accuracy of stool-based tests for CRC screening.
• Our results showed that both Mt-sDNA panel and the FIT test had acceptable cut-off points for cancer detection.
• Among four stool -based tests, Mt-sDNA test had the highest sensitivity for detection of cancer and advanced adenoma.

## 1 Background

Although the incidence of colorectal cancer varied in different parts of the world, according to the latest report of the International Agency for Research on Cancer in 2020, colorectal cancer is the third most common cancer and the second leading cause of death from cancer in the world [1]. Over recent years there were improvements in incidence and mortality of colorectal cancer globally, attributed to early detection and screening programs [2]. However, several epidemiological studies suggest that even though Iran is still in a low-risk region of colorectal cancer, especially in older populations, the incidence of colorectal cancer is increasing compared to previous reports [[3], [4], [5], [6], [7]]. Currently, colorectal cancer is the third most common cancer in men and the second common cancer in women of all ages in Iran [8]. In addition, colorectal cancer is currently the third most common cancer in both genders in East Azerbaijan province of Iran and its incidence is increasing during the last decade [9,10].

Studies have shown that both incidence and mortality from colorectal cancer can be reduced and prevented using screening programs and early withdrawal of any polyp and primary lesion [11]. However, colorectal cancer screening is still underused, even in the most developed countries. The most presented barriers were invasiveness of tests, complications' risks, and some difficulties and inconvenience with colonoscopy as the most often used modality; therefore, noninvasive, accessible, and easy to use methods were provided and implemented. Nowadays, stool-based tests are good alternative choices with suboptimal sensitivity for early detection of colorectal cancer [12]. High sensitivity gFOBT and fecal immunochemical tests (FIT) are specific for detecting human hemoglobin in stool samples, however, despite improved analytical and clinical sensitivity for cancer detection, colonoscopy remains the recommended screening modality with better diagnostic accuracy [13,14].

The milestone of using mt-sDNA testing has commenced since 2009 which has been co-developed by Mayo Clinic and approved by the US Food and Drug Administration (FDA) in August 2014 [11]. The final approved panel named “Cologuard” (Exact Sciences, Madison, WI, USA) was included DNA mutation (KRAS, BRAF), DNA methylation (BMP-3, NDRG-4), and fetal hemoglobin immunoassay and DNA β-actin, for clinical assay development [15,16]. According to the last released guidelines of the American Cancer Society in 2018, U.S. Preventive Services Task Force in 2016, and the National Comprehensive Cancer Network in 2016, multi-target stool DNA (Mt-sDNA) tests (combined FIT-DNA stool tests) every three years have been recommended as a potential screening method in average-risk populations, with almost the same results in CRC incidence reduction compared with 10-year colonoscopy (63% vs. 65%) [[17], [18], [19]].

The current research study aimed to assess the sensitivity and specificity of stool-based tests compared with colonoscopy, in the Northwest population of Iran. The primary outcome was the sensitivity and specificity of mt-sDNA for colorectal cancer compared with colonoscopy, the secondary outcome was the sensitivity of all tests for advanced adenoma. Specificity was calculated using normal colonoscopy results (without cancer and AA).

## 2 Methods

### 2.1 Aim, design and setting of the study

The current study has been designed as a cross-cultural analytic study, which aimed toassess the feasibility of the colorectal cancer screening program in East Azerbaijan Province and to evaluate the diagnostic accuracy of stool-based tests compared with colonoscopy, as the first step to provide the best CRC screening modality in our population. East Azerbaijan is a province located in the Northwest of Iran, which has the sixth largest population with the most Azeri ethnic population in Iran.

### 2.2 Characteristics of participants

From March 2016 to February 2019, 200 cases were evaluated. From these, 44 cases were excluded due to their missing stool samples and colonoscopy results. Then, 156 cases were eligible for our study, after providing informed consent forms.

The “CRC Risk Assessment Questionnaire” was completed for all recruited subjects developed to assess the CRC risk based on personal and family history of adenoma, CRC, and inflammatory bowel disease (ulcerative colitis, Crohn's disease). Demographic information including age, gender, and race/ethnicity, positive family history with detailed pedigree has been assessed.

### 2.3 Inclusion and exclusion criteria

The eligibility criteria were based on CRC Risk Assessment tool. This tool was the translated and validated NCCN guideline which included a questionnaire consisting of simple and easy-to-use questions about the main risk factors of CRC [20]. Research team staff assessed all individuals by this tool and then eligible people according to their lifetime risk of colorectal cancer were included in the study, including:

Average risk: Individuals with a negative personal and family history of adenoma, polyps, CRC, or inflammatory bowel disease, and who were aged ≥50 years;

Increased risk: Individuals of any age with a personal history of adenoma, polyps, CRC, or inflammatory bowel disease, and those with a positive family history of CRC or with high-grade adenomatous polyps;

High-risk syndrome: Individuals with a family history of hereditary non-polyposis colorectal cancer (HNPCC-1 or HNPCC-2) or with a personal or family history of polyposis syndrome.

We excluded any individuals whom;

- Regret to continue to all screening modalities in the study
- Regret or fear of having a colonoscopy
- Having other cancer and/or underlying chronic diseases

### 2.4 Description of processes, interventions and comparisons

For assessing the mass screening methods for colorectal cancer, the main recommended methods including colonoscopy (as the golden standard) and stool-based test were performed for all participants. Colonoscopy was performed in 4 referral hospitals, by 10 expert gastroenterologists. The colonoscopy results have been tracked and recorded, and in the case of any biopsies of suspected lesions during the procedure, the pathological results were assessed and recorded. Stool-based tests were performed in Hematology and Oncology and Immunology research centers of Tabriz University of Medical Sciences, including traditional guaiac-based fecal occult blood test (gFOBT), high-sensitivity guaiac-based fecal occult blood test (gFOBT Hb), fecal immunochemical test (FIT), and Mt-sDNA panel test.

### 2.5 Molecular tests

The multitarget stool DNA test consisted of molecular assays for mutant KRAS and BRAF, aberrantly methylated BMP3 and NDRG4 promoter regions, including aberrant methylation in the promoter regions of the “NDRG4” gene (N-Myc Downstream-Regulated Gene 4) and “BMP3” gene (Bone Morphogenetic Protein 3 gene), and β-actin (a reference gene for human DNA quantity).

#### 2.5.1 DNA extraction

DNA was extracted from stool samples, using QIAamp® DNA Stool Mini Kit (50) (QIAgene, cat. no. 51504), according to the kit guideline.

To assess the methylation state, the extracted DNA must be treated with sodium bisulfite. This treatment results in the conversion of the un-methylated cytosine to uracil. In the current study, EZ DNA Methylation Kit (Zymo Research, cat. no. D5001) was used for this purpose.

#### 2.5.2 Evaluating the mutations of KRAS and BRAF genes

Mutations mostly in Codon 12 and 13 on exon 2 of the KRAS gene and exon 15 of BRAF gene (leading to the substitution of glutamine for valine at codon 600, V600E) are among the most reliable prognostic factors for susceptibility to CRC.

In current study, High- Resolution Melt (HRM) analysis was employed to assess the mutation status of the mentioned genes. This method costs much lower than sequencing of the whole product but shown to have acceptable accuracy. For this purpose a few pairs of primers were designed and, based on their performance, the best primer pair was selected for evaluating KRAS and BRAF mutations (Table 1, Table 2).

**Table 1:** Primer and conditions used to investigate KRAS gene mutations.

Primer	F: GTTTGTATTAAAAGGTACTGGTGG R: CTGAATTAGCTGTATCGTCAAGG			
Product Length	170bp			
PCR Cycling	Stage	Temperature	Time	Repeat No.
	Pre- incubation	94 C	600s	1
	Denaturation	94 C	15s	45
	Annealing	60 c	30s	
	Extension	72 C	20	
	Final Extension	72 C	300 s	1
	HRM	Equipment default		−1

**Table 2:** Primer and conditions used to investigate BRAF gene mutations.

Primer	F:TCATGAAGACCTCACAGTAAAAATAGG-3′ R:5′-AGCAGCATCTCAGGGCCAAA-3′			
Product Length	164bp			
PCRCycling	Stage	Temperature	Time	Repeat Number
	Pre- incubation	94 C	600s	1
	Denaturation	94 C	15s	45
	Annealing	59 c	30s	
	Extension	72 C	20	
	Final Extension	72 C	300 s	1
	HRM	Equipment default		−1

#### 2.5.3 METH-HRM method for detecting NDRG-4 and BMP-3 methylation status

The MS-HRM method was employed to assess the methylation status of NDRG-4 and BMP-3 genes in current study. Using meth-primer software, 4 pairs of primers were designed for assessing methylation in CpG islands. After the experimental evaluation, the best primer pairwise was selected for each gene (Table 3, Table 4).

**Table 3:** Primer sequences and PCR conditions for investigation of BMP-3 gene methylation.

Primer sequence	TTTAGTTTGGTGTAAGTTAAGAGGG CTTTTCCAAAAATTAAAACAACTAC			
Product Length	141			
PCR conditions	Stage	Temperature	Time	Repeat Number
	Pre- incubation	94 C	600s	1
	Denaturation	94 C	15s	45
	Annealing	60 c	30s	
	Extension	72 C	20	
	Final Extension	72 C	600s	1
	HRM	Equipment default-		−1

**Table 4:** Primer sequences and PCR conditions for investigation ofNDRG-4gene methylation.

Primer sequences	AGGGTTTTTGTTTTTTAATTAGTGT AATCCAATCTAACTTCCCACTTC			
Product Length	161			
PCR conditions	Stage	Temperature	Time	Repeat Number
	Pre- incubation	94 C	600s	1
	Denaturation	94 C	15s	45
	Annealing	60 c	30s	
	Extension	72 C	20	
	Final Extension	72 C	600s	1
	HRM	- Equipment default-		−1

## 3 Statistical analyses

The main recommended method including colonoscopy (as golden standard), and stool-based tests were evaluated for sensitivity, specificity, Positive Predictive Value (PPV), Negative Predictive Value (NPV), positive likelihood ratio (LR+), and negative likelihood ratio (LR-) with 95% confidence interval using STATA 12 software (StataCorp, College Station, Texas, USA).

A positive likelihood ratio (LR+) is the probability of a positive outcome given a positive screening. The absolute value of the LR + represents the magnitude of the probability with LR+ of 1–2 indicating minimal probability; 2–5 indicating small probability; 5–10 indicating moderate probability, and>10 indicating large and conclusive probability. A negative likelihood ratio (LR-) is the probability of a negative outcome given a negative screening. LR-from 0.0 to 0.2 provides relatively high probability, 0.2–0.5 represents a moderate probability, 0.5–1.0 is interpreted to mean there is a minimal probability.

ROC analysis was performed to assess the relationship between clinical sensitivity and specificity and for the equality of tests compared with colonoscopy, as the areas under ROC curves are used to compare the usefulness of tests, where a greater area means a more useful test. The range AUC ≥0.8 represents the high probability, AUC ≥0.7 represents the moderate probability, and AUC ≤0.5 was unacceptable.

## 4 Results

Overall 156 cases were recruited in current study, including 99 (63%) males and 58 (34%) females. From these 121 cases (77%) were ≥50 years old, and 36 cases (23%) were <50 years old. Of 156 participants, 9 (5.77%) had CRC, and 9 (5.77%) cases had AA, and 138 (88.46%) cases had a normal colonoscopy. From 9 CRCs, 3 cases had cancer in the ascending colon, and 6 in the descending colon. From 9 AA, two of them were in the ascending colon and 7 in the descending colon (Table 5).

**Table 5:** Summary Statistics of 156 eligible participants.

		Age		Gender	
	Number (%)	<50	≥50	Male	Female
Colorectal Cancer	9 (5.77)	5 (55.55)	4 (44.45)	3 (33.33)	6 (66.67)
Advanced Adenoma	9 (5.77)	3 (33.33)	6 (66.67)	3 (33.33)	6 (66.67)
Normal	138 (88.46)	27 (19.57)	111 (80.43)	76 (55.07)	62 (44.93)
Total	156	35 (22.44)	121 (77.56)	82 (52.56)	74 (47.44)

### 4.1 Results of colonoscopy

At the first step, all candidates were undergoing colonoscopy as a golden standard for colorectal cancer detection. According to the colonoscopy results, 18 individuals were found to have abnormalities in their colorectal tissues whose samples were sent for pathological assessments to identify their exact problems. The Results of pathology analysis revealed that 9 (5.77%) had CRC and 9 (5.77%) of the subjects had advanced adenoma.

### 4.2 Stool based tests

In addition to colonoscopy, the presence of blood in stool samples was evaluated using three different methods including traditional FOB Guaiac, high sensitivity FOB Hb and FIT. Due to our results, FOB Guaiac was positive in 8 samples and among them, 5 samples were positive for colorectal cancer. High sensitivity FOB Hb was positive in 8 samples, and 6 cases had confirmed CRC. The FIT was positive in 8 samples and due to pathological results, 6 of them were malignant as well. Among 10 samples positive detected with Mt-sDNA panel, 7 were positive as confirmed CRC.

### 4.3 K-RAS, B-RAF mutations

**Fig. 1:**
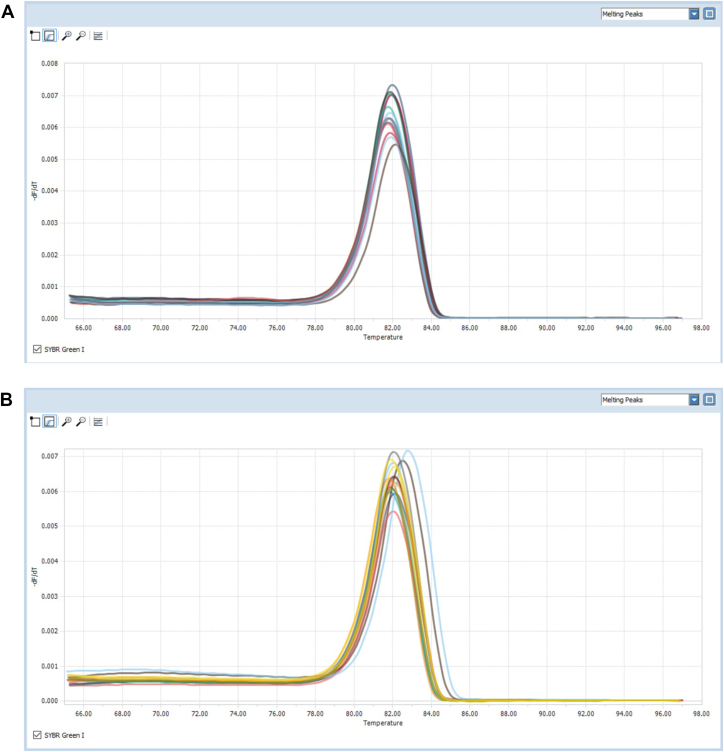
Melting diagram of (A) KRAS gene, (B) BRAF gene.

HRM were employed to detect K-RAS and B-RAF mutations and we could not detect any mutation in our samples (Fig. 1).

### 4.4 Methylation of NDRG-4 and BMP-3

**Fig. 2:**
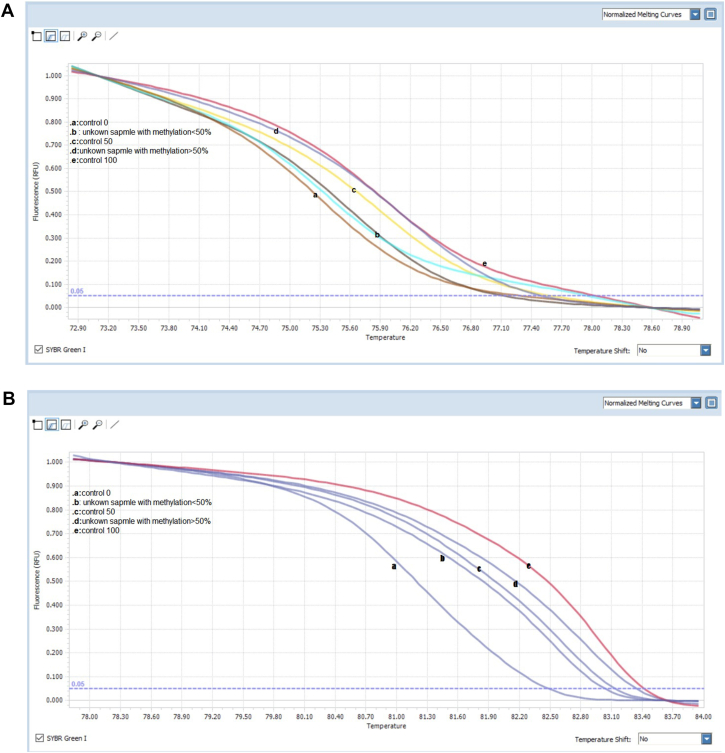
Methylation diagram of (A) NDRG-4, (B) BMP-3 promoters.

Due to our results, 7 samples were hypermethylated in the NDRG-4 promoter which 5 of them were positive for malignancy (sensitivity = 0.63 and specificity = 0.97). BMP-3 promoter was hypermethylated in 5 samples and 4 of them were positive for colorectal cancer (sensitivity = 0.36 and specificity = 0.99). Simultaneous hypermethylation of NDRG-4 and BMP-3 promoters was common in only one malignant sample (Fig. 2).

### 4.5 Diagnostic accuracy findings

Mt-sDNA test had the highest diagnostic accuracy ratios, with a sensitivity of 77.8% (95% CI: 40–97.2) for the detection of CRC, and 33.3% (95% CI: 7.49–70.1) for the detection of AA, with a combined specificity of 92.8% (95% CI: 87.1–96.5) for the detection of CRC and AA. The FIT test alone had a sensitivity of 66.7% (95% CI: 29.9–92.5), and 22.2% (95% CI: 2.81–60) for the detection of CRC and AA respectively, with a combined specificity of 94.9% (95% CI: 89.8–97.9).

The likelihood ratio of Mt-sDNA test had a moderate probability for detection of CRC (LR+ = 8.79; 95% CI: 4.7–16.4), with a LR negative = 0.24 (95% CI: 0.07–0.83). Positive predictive value (PPV) of Mt-sDNA test was lower than that of the FIT test alone for the detection of CRC (35% vs 40%), but the combined negative predictive value of Mt-sDNA test was higher than that of other tests NPV = 94.1%; 95% CI: 88.7–97.4) (Table 6).

**Table 6:** Diagnostic Accuracy Analysis Results of Mt-sDNA, FIT, high sensitivity FOB Hb, and traditional FOB Guaiac test compared with Colonoscopy (as golden standard method).

		TP^a^	Sensitivity % (95% CI)	Specificity % (95% CI)	ROC^h^ area (95% CI)	LR +^b^ (95% CI)	LR -c (95% CI)	PPV %^d^ (95% CI)	NPV %^e^ (95% CI)
Mt-sDNA test	CRC^f^ (n = 9)	7	77.8 (40–97.2)	91.2 (85.4–95.2)	0.85 (0.69–0.99)	8.79 (4.7–16.4)	0.24 (0.07–0.83)	35 (15.4–59.2)	98.5 (94.8–99.8)
	AA^g^ (n = 9)	3	33.3 (7.49–70.1)	88.4 (82.1–93.1)	0.61 (0.44–0.77)	2.88 (1.03–8.04)	0.75 (0.47–1.2)	15 (3.21–37.9)	95.6 (90.6–98.4)
	Both (n = 18)	10	55.6 (30.8–78.5)	92.8 (87.1–96.5)	0.74 (0.62–0.86)	7.67 (3.71–15.8)	0.48 (0.29–0.81)	50 (27.2–72.8)	94.1 (88.7–97.4)

FIT	CRC (n = 9)	6	66.7 (29.9–92.5)	93.9 (88.7–97.2)	0.80 (0.64–0.97)	10.9 (4.97–23.8)	0.36 (0.14–0.89)	40 (16.3–67.7)	97.9 (93.9–99.6)
	AA (n = 9)	2	22.2 (2.81–60)	91.2 (85.4–95.2)	0.57 (0.42–0.71)	2.51 (0.67–9.48)	0.85 (0.6–1.21)	13.3 (1.66–40.5)	95 (90–98)
	Both (n = 18)	8	44.4 (21.5–69.2)	94.9 (89.8–97.9)	0.69 (0.58–0.82)	8.76 (3.61–21.3)	0.59 (0.39–0.89)	53.3 (26.6–78.7)	92.9 (87.3–96.5)

FOB Hb^i^	CRC (n = 9)	6	66.7 (29.9–92.5)	95.2 (90.4–98.1)	0.81 (0.65–0.97)	14 (5.94–33)	0.35 (0.14–0.88)	46.2 (19.2–74.9)	97.9 (94–99.6)
	AA (n = 9)	2	22.2 (2.81–60)	92.5 (87–96.2)	0.57 (0.43–0.72)	2.97 (0.77–11.4)	0.84 (0.59–1.2)	15.4 (1.92–45.4)	95.1 (90.2–98)
	Both (n = 18)	8	44.4 (21.5–69.2)	96.4 (91.7–98.8)	0.70 (0.59–0.82)	12.3 (4.5–33.5)	0.58 (0.38–0.87)	61.5 (31.6–86.1)	93 (87.5–96.6)

FOB Guaiac^j^	CRC (n = 9)	5	55.6 (21.2–86.3)	91.2 (85.4–95.2)	0.73 (0.56–0.91)	6.28 (2.88–13.7)	0.49 (0.23–1.01)	27.8 (9.69–53.5)	97.1 (92.7–99.2)
	AA (n = 9)	3	33.3 (7.49–70.1)	89.8 (83.7–94.2)	0.62 (0.45–0.78)	3.27 (1.15–9.25)	0.74 (0.47–1.18)	16.7 (3.58–41.4)	95.7 (90.8–98.4)
	Both (n = 18)	8	44.4 (21.5–69.2)	92.8 (87.1–96.5)	0.69 (0.57–0.81)	6.13 (2.79–13.5)	0.59 (0.39–0.91)	44.4 (21.5–69.2)	92.8 (87.1–96.5)

a True Positive.

b Positive Likelihood Ratio.

c Negative Likelihood Ratio.

d Positive Predictive Value.

e Negative Predictive Value.

f Colorectal Cancer.

g Advanced Adenoma.

h ROC, receiver operating characteristic.

i Traditional guaiac-based fecal occult blood tests.

j High sensitivity guaiac-based fecal occult blood tests.

**Fig. 3:**
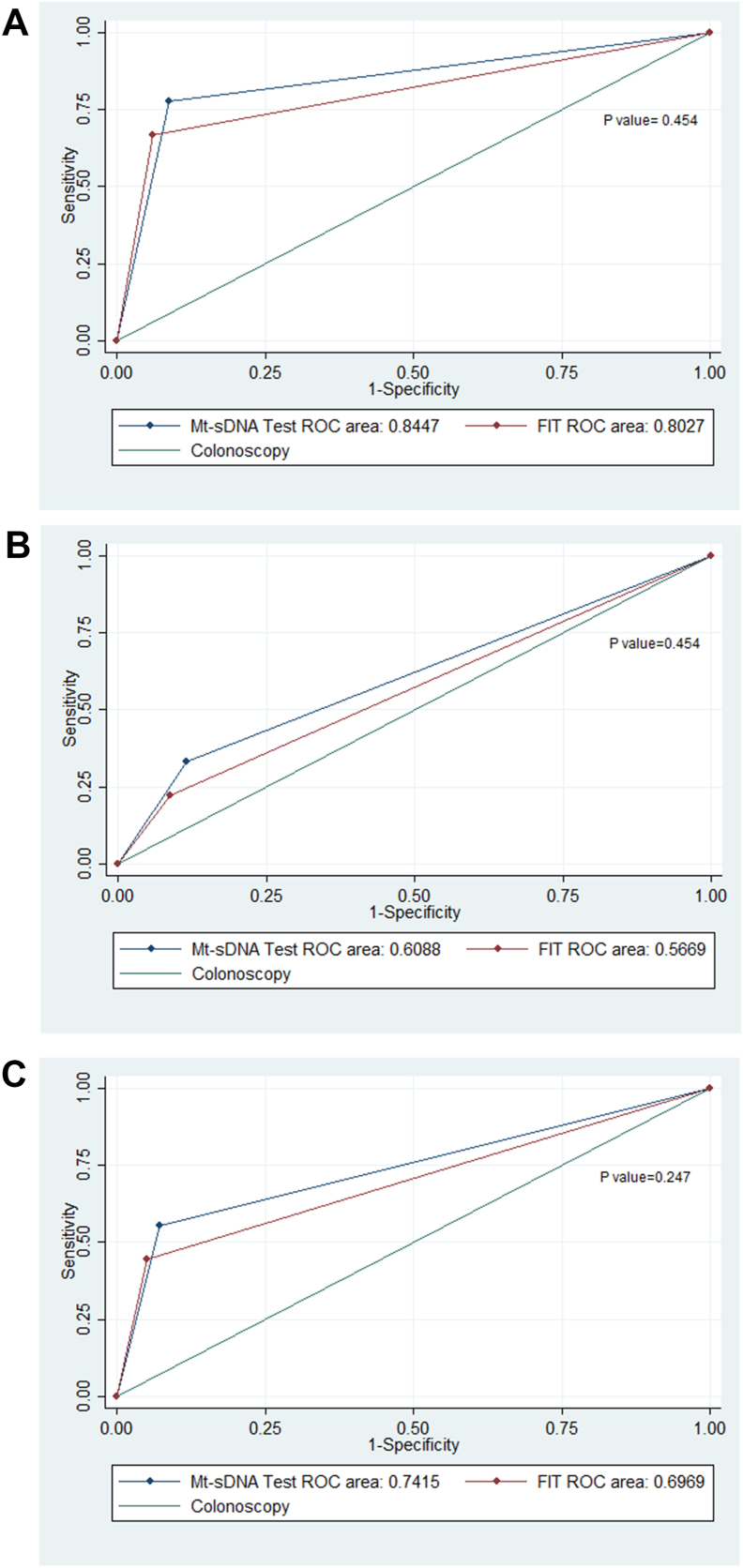
ROC curves of diagnostic accuracy of Mt-sDNA panel and the FIT test for (A) CRC, (B) AA, and (C) combined CRC and AA (AUC, area under the receiver operating characteristic ROC).

**Fig. 4:**
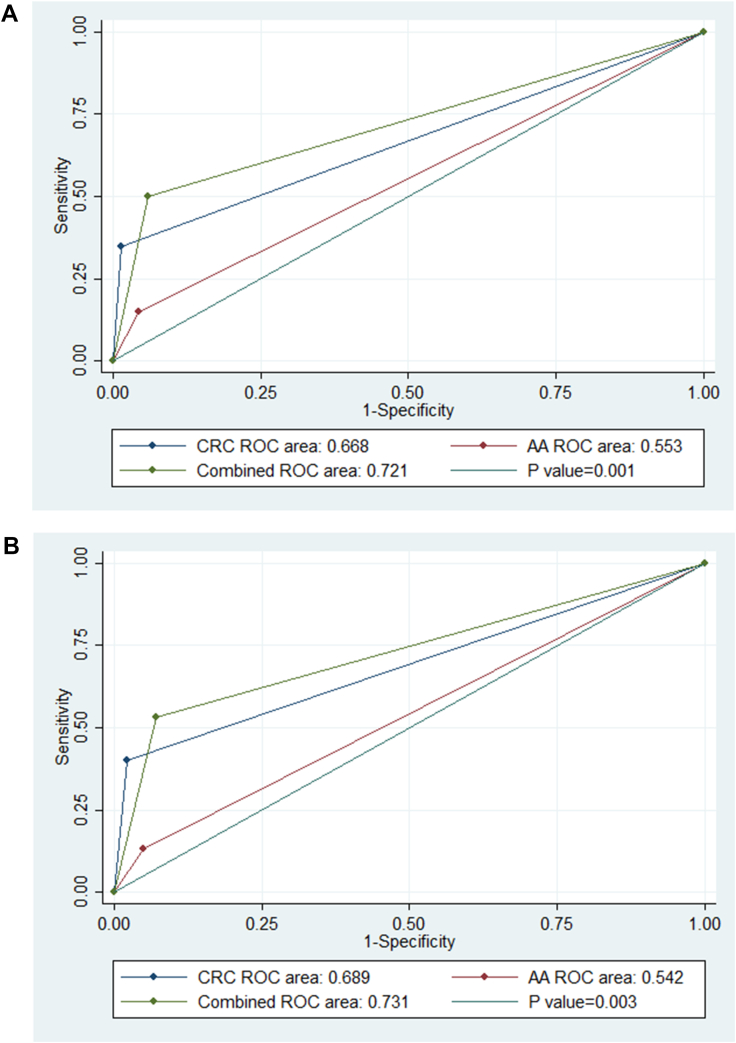
ROC curves of the diagnostic accuracy of CRC, AA, and combined for (A) Mt-sDNA panel (B) FIT test (AUC, area under the receiver operating characteristic ROC).

The test equality of ROC area analysis showed that both Mt-sDNA and the FIT tests alone had an acceptable ROC range (0.85 vs 0.80) for the detection of CRC compared with colonoscopy (P = 0.454). Also, ROC area analysis for combined CRC and AA detection was more acceptable for Mt-sDNA test (ROC area = 0.74; 95% CI: 0.62–0.86), than the FIT test alone (ROC area = 0.69; 95% CI: 0.58–0.82) (P = 0.247) (Fig. 3). ROC area analysis showed a statistically significant difference between the diagnostic accuracy of both Mt-sDNA and the FIT tests alone for diagnosis of CRC, AA, and combined CRC and AA (P value = 0.001 vs. P value = 0.003) (Fig. 4).

## 5 Discussion

The current study was conducted in the Northwest of Iran as a feasibility study of a screening program of colorectal cancer and setting the new Mt-sDNA test in the region. Also, we tried to evaluate the diagnostic accuracy of Mt-sDNA test panel, the FIT, and gFOBT tests compared with colonoscopy, as the first step to provide the best CRC screening modality in our population. All individuals were first assessed by our established CRC risk assessment tool, then eligible volunteers from average and high-risk groups and relative ages entered the study. Colonoscopy was performed for all participants, also stool-based tests including traditional guaiac, high-sensitivity guaiac-based, the FIT and Mt-sDNA panel tests were performed.

According to the results of the current study, Mt-sDNA test panel had a sensitivity of 77.8% for the detection of colorectal cancer with a specificity of 91.2%. The FIT test alone had a lower sensitivity (66.7%) and almost the same specificity of 93.9% for cancer detection. Also, ROC analytic results showed that both Mt-sDNA panel and the FIT test had acceptable cut-off points for cancer detection, however, Mt-sDNA test had better diagnostic accuracy than the FIT (AUC = 0.85 vs 0.80), and is a more useful screening test. The positive likelihood ratio for Mt-sDNA test was in a large and conclusive range (LR+ 8.79), which indicated the absolute probability of cancer detection by Mt-sDNA test. Negative likelihood ratio test which shows the probability of negative cases for cancer was 0.24 for Mt-sDNA, which was relatively in the high probability range. The LR+ was higher for the FIT test alone with an LR+ of 10.9, but the FIT test alone had a moderate probability for the negative outcome (LR- 0.36). Positive and negative predictive values for cancer detection for both Mt-sDNA and the FIT tests had almost the same results, however Mt-sDNA test had better NPV results than the FIT test alone for cancer, AA, and combined CRC and AA.

Comparing these results with the last published NCCN guideline for colorectal cancer screening revealed interesting results (Table 7). The first colonoscopy still has the highest sensitivity for CRC (95%) and AA (89–98%), with a specificity of 90%. The last provided results of diagnostic accuracy of other stool-based tests are comparable with our results, while in this guideline Mt-sDNA test had a sensitivity of 92% for CRC (vs our results: 91.2%), 42% for AA (vs our results: 33.3%), with a specificity of 87% (vs our results: 92.8%). Although the FIT was still more specific for both CRC and AA in both results which indicates that the FIT had fewer false-positive results, we are aware that sensitivity is the most important characteristic of any screening modality [17].

**Table 7:** Comparing Diagnostic Accuracy Results with the last published NCCN guideline for colorectal cancer screening.

	Sensitivity				Specificity	
	Colorectal Cancer		Advanced Adenoma			
	NCCN Guideline	Our Results	NCCN Guideline	Our Results	NCCN Guideline	Our Results
high-sensitivity guaiac-based test	62–79%	66.7%	7%	22.2%	87–96%	96.4%
FIT	76–95%	66.7%	27–47%	22.2%	89–96%	94.9%
Mt-sDNA panel	92%	91.2%	42%	33.3%	87%	92.8%

Despite wide use of colonoscopy in many countries as the golden standard for CRC screening and early diagnosis, it has some main disadvantages which play the role of the predominant barriers and limitations for implementation on a large scale in different populations [21]. Missed diagnosis of AA, side-effecting detection biases, fear and discomfort related to colonoscopy, bowel preparation difficulties, and possible harms have been presented as the predominant barriers [[22], [23], [24]]. Both Mt-sDNA and the FIT tests are noninvasive and patient-friendly, have acceptable diagnostic accuracy do not need any food and drug restrictions, and have higher adherence in the younger age group and average-risk population [11,16,25]. Despite the advantages and harmless of stool-based tests, colonoscopy is still the most accepted and available screening modality in numerous countries, with the advantage of higher sensitivity, ability to remove pre-cancerous lesions and with a longer rescreening interval [2,16,19,24]. Although current guidelines provided using non-invasive screening modalities, it has been strongly suggested that all cases with positive stool-based tests should undergo a visual colonoscopy to detect and remove any precancerous lesions [26,27].

Familial colorectal cancer accounts for 35% of CRC cases, however, 85% of sporadic CRCs have molecular changes like chromosomal instability (CIN), microsatellite instabilities (MSI). However, colorectal cancer has diverse pathogenesis, with different molecular changes and affected pathways [3,28,29]. Therefore, biomarkers recently received important attention for using “in screening and early detection programs” of colorectal cancer [30]. Since the approval of Mt-sDNA test as screening modality in CRC screening from US Food and Drug Administration (FDA) in 2014, there was an increase in the use of this test by providers and patients [11,16]. Most recently, the development of next-generation mt-sDNA testing with higher specificity, has led to detecting at least 3 novel DNA methylated markers [11,16,31].

Most recently Imperial et al., conducted a prospective cross-sectional study on 983 participants to determine the specificity of the multitarget stool DNA test in younger volunteers (45–49 YO) and the average-risk population. They revealed that Mt-sDNA panel had higher specificity (95.2%) in this age group, even better than colonoscopy and the FIT [2]. Recently, US Preventive Services Task Force endorsed Mt-sDNA test as a first-line CRC screening modality, by focusing on use in patients 45–49 years old [11]. The lower prevalence of any precancerous lesion and CRC in younger and average age groups leads to higher specificity, which means lower false-positive cases [11,16,18]. While according to last released screening guidelines from the American Cancer Society (ACS) which recommend commencing CRC screening from age 45, these pieces of evidence should be taken in mind in terms of advantages of noninvasive, higher specificity and sensitivity and better acceptance in young age groups [2].

The superior sensitivity of Mt-sDNA tests is still under fire because of its high cost and poor availability, and it is still equivocal how test results could be corroborated independently by other tests. However, recently cost-effective analysis revealed that colorectal cancer screening with triennial Mt-sDNA test reduced significantly CRC incidence and mortality compared with no screening; however, was the most costly and less available strategy compared to colonoscopy and/or the FIT tests [32]. There is still a need for larger studies and screening trials with evidence-based analyses to prove the benefits of this test, despite its cost and availability.

Recently CRC screening recommended commencing at age 45, according to the American Cancer Society, and this may be implemented in Iran as well [11,33]. Overall, developing a comprehensive strategy for providing the best screening method and the best age for screening for colorectal cancer in Iran is strongly recommended. However, the design and implementation of screening programs in each community depend on identifying average and high-risk people, and launching a primary risk assessment tool.

Despite available evidence-based guidelines for colorectal cancer screening, for establishing an effective screening program in each population several joint studies and pieces of evidence should be considered., therefore, we are trying to introduce a suitable screening program to be used in the country Consequently, several factors are considered to determine whether the benefits of screening modality outweigh the risks and costs of the tests and according to WHO recommendations, these factors will include:

• Possible harms from the screening test
• The likelihood of the test correctly identifying cancer
• The likelihood of colorectal cancer
• Possible harms from follow-up procedures
• Whether suitable treatment is available and appropriate
• Whether early detection improves treatment outcomes
• Whether the cancer will ever need treatment
• Whether the test is acceptable to the patients
• Cost of the test
• The extent to which a cancer is treatable

## 6 Conclusion

The current study has been designed as a cross-cultural analytic study, and to evaluate the diagnostic accuracy of stool-based tests compared with colonoscopy, as the first step to provide the best CRC- screening modality in our population during 2016–2020. Our results showed that both Mt-sDNA panel and the FIT test had acceptable cut-off points for cancer detection, however, Mt-sDNA test had better diagnostic accuracy than the FIT, and is a more useful screening test. Among four stool -based tests, Mt-sDNA test had the highest sensitivity for detection of cancer and advanced adenoma. Our results of the diagnostic accuracy of stool-based tests for CRC screening was comparable with the recently provided guidelines.

## Ethical approval

Current study was reviewed and corroborated by the ethics committee of Tabriz University of Medical Sciences (ID: IR.TBZMED.REC.1395.1333). Ethical consents were obtained from all the participants and all information and results were confidential.

## Funding

The study was approved and supported as a research grant, by Ministry of Health and Medical Education, Deputy of Research and Technology, Iran (Grant number: 700/98, 2015.03.14 [1394/12/24]). This fund supported the research study methods and performed tests, and any support for manuscript writing.

## Author contributions

RD, MHS: Performed drafting the work; supervised the research project and work; and wrote the first draft of work; and revised manuscript critically for important intellectual content;

RD, SD, and MAJ: Performed substantial contributions to the conception and design of the work; the acquisition, analysis, and interpretation of data for the work;

DS, MA: Performed methodological and molecular work; and interpretation of data and results; FF, ND, MHS: Revised and approved the version to be published.

## Registration of research studies

Hyperlink to your specific registration (must be publicly accessible and will be checked): N/A.

Unique Identifying number or registration ID: IR.TBZMED.REC.1395.1333.

Name of the registry: Tabriz University of Medical Sciences.

## Consent for publication

All the authors confirmed agreement to be accountable for all aspects of the work in ensuring that questions related to the accuracy or integrity of any part of the work are appropriately investigated and resolved.

The manuscript has been read and approved by all the authors, that the requirements for authorship as stated earlier in this document have been met, and that each author believes that the manuscript represents honest work, if that information is not provided in another form.

## Consent

Ethical consents were obtained from all the participants and all information and results were confidential.

## Guarantor

Roya Dolatkhah, Hematology and Oncology Research Center, Tabriz University of Medical Sciences, Tabriz, Iran, Email: dolatkhahr@tbzmed.ac.ir, royadolatkhah@yahoo.com.

## Availability of data and materials

The datasets generated during and/or analyzed during the current study are available from the corresponding author on reasonable request.

## Provenance and peer review

Not commissioned, externally peer reviewed.

## Declaration of competing interest

The authors have no relevant financial or non-financial interests to disclose.
